# 1,3-Diallyl-1*H*-anthra[1,2-*d*]imidazole-2,6,11(3*H*)-trione

**DOI:** 10.1107/S1600536810024748

**Published:** 2010-06-26

**Authors:** Zahra Afrakssou, Youssef Kandri Rodi, Hafid Zouihri, El Mokhtar Essassi, Seik Weng Ng

**Affiliations:** a Laboratoire de Chimie Organique Appliquée, Faculté des Sciences et Techniques, Université Sidi Mohamed Ben Abdallah, Fès, Morocco; b CNRST Division UATRS, Angle Allal Fassi/FAR, BP 8027 Hay Riad, Rabat, Morocco; c Laboratoire de Chimie Organique Hétérocyclique, Pôle de Compétences Pharmacochimie, Université Mohammed V-Agdal, BP 1014 Avenue Ibn Batout, Rabat, Morocco; d Department of Chemistry, University of Malaya, 50603 Kuala Lumpur, Malaysia

## Abstract

In the title compound, C_21_H_16_N_2_O_3_, the fused-ring system (r.m.s. deviation = 0.067 Å) is slightly buckled at the carbonyl C atom of the anthracenyl ring system [deviation = 0.177 (1) Å] that is closer to an allyl substituent. The two allyl units lie on the same side of the fused-ring plane but are oriented in opposite directions, with N—C—C—C torsion angles of 126.9 (2) and 116.7 (2)°. In the crystal, the mol­ecules are linked into chains propagating along the *b* axis by C—H⋯O hydrogen bonds.

## Related literature

For a related structure, see: Guimarães *et al.* (2009 ▶).
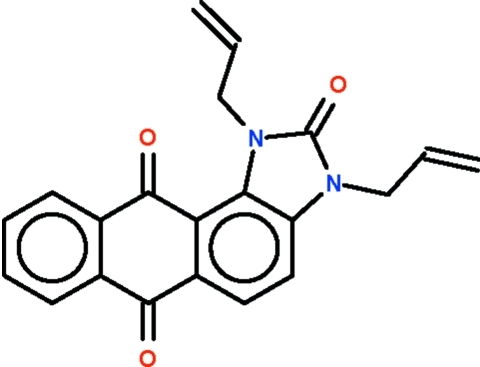


## Experimental

### 

#### Crystal data

C_21_H_16_N_2_O_3_
*M* *_r_* = 344.36
Monoclinic, 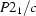

*a* = 7.8539 (2) Å
*b* = 11.5822 (3) Å
*c* = 18.1455 (4) Å
β = 93.537 (1)°
*V* = 1647.47 (7) Å^3^
*Z* = 4
Mo *K*α radiation
μ = 0.09 mm^−1^
*T* = 293 K
0.40 × 0.35 × 0.20 mm

#### Data collection

Bruker X8 APEXII area-detector diffractometer
22612 measured reflections
4806 independent reflections 4805 in *Refinement*?
3053 reflections with *I* > 2σ(*I*)
*R*_int_ = 0.039

#### Refinement

*R*[*F* ^2^ > 2σ(*F* ^2^)] = 0.049
*wR*(*F* ^2^) = 0.153
*S* = 1.02
4805 reflections
236 parameters
H-atom parameters constrained
Δρ_max_ = 0.31 e Å^−3^
Δρ_min_ = −0.22 e Å^−3^

### 

Data collection: *APEX2* (Bruker, 2008 ▶); cell refinement: *SAINT* (Bruker, 2008 ▶); data reduction: *SAINT*; program(s) used to solve structure: *SHELXS97* (Sheldrick, 2008 ▶); program(s) used to refine structure: *SHELXL97* (Sheldrick, 2008 ▶); molecular graphics: *X-SEED* (Barbour, 2001 ▶); software used to prepare material for publication: *publCIF* (Westrip, 2010 ▶).

## Supplementary Material

Crystal structure: contains datablocks global, I. DOI: 10.1107/S1600536810024748/ci5112sup1.cif

Structure factors: contains datablocks I. DOI: 10.1107/S1600536810024748/ci5112Isup2.hkl

Additional supplementary materials:  crystallographic information; 3D view; checkCIF report

## Figures and Tables

**Table 1:** Hydrogen-bond geometry (Å, °)

*D*—H⋯*A*	*D*—H	H⋯*A*	*D*⋯*A*	*D*—H⋯*A*
C13—H13⋯O3^i^	0.93	2.49	3.406 (2)	168
C16—H16*B*⋯O3^i^	0.97	2.42	3.362 (2)	165

Symmetry code: (i) 
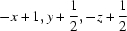
.

**Fig. 1.**
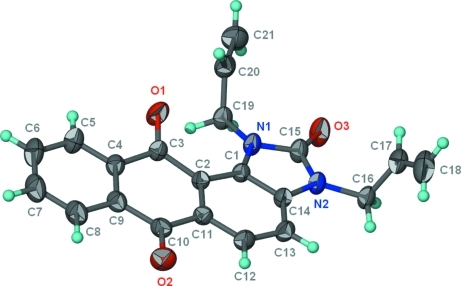
Displacement ellipsoid plot (Barbour, 2001) of C21H16N2O3 at the 50% probability leve. H atoms are drawn as spheres of arbitrary radii.

## supplementary crystallographic information

### Comment

An imidazol-one such as 1*H*-anthra[2,1-*d*]imidazole-2,6,11(3*H*)-trione, in which the five-membered ring is fused with an anthraquinone system, alkyl halides under catalytic conditions to yield di-*N*,*N*'-substituted derivatives that serve as starting reagents for the synthesis of other drugs. The anthraquinone system itself is found in a large number of pigments and dyes. The title compound (Scheme I, Fig. 1) is a deep orange material that may be useful as an organic fluorophone.

The title molecule features four rings that are fused together (r.m.s. deviation 0.067 Å). The fused-ring system is slightly buckled at that carbonyl C-atom, C3, of the anthracenyl system [0.177 (1) Å] that is closer to an allyl substituent. The pendant allyl units lie on the same side of the fused-ring plane but are oriented in opposite directions. The crystal packing is stabilized by C—H···O hydrogen bonds (Table 1).

### Experimental

To a solution of 1*H*-anthra[2,1-*d*]imidazole-2,6,11(3*H*)-trione (1.00 g, 0.38 mmol), potassium carbonate (1.56 g,11 mmol) and tetra *n*-butyl ammonium bromide (0.12 g, 0.38 mmol) in DMF (20 ml)) was added allyl bromide (0.77 ml, 11 mmol). Stirring was continued at room temperature for 24 h. The mixture was filtered and the solvent removed. The residue was extracted with water. The organic compound was chromatographed on a column of silica gel with ethyl acetate-hexane (1/1) as eluent. Orange crystals were isolated when the solvent was allowed to evaporate.

### Refinement

H atoms were placed in calculated positions (C–H = 0.93–0.97 Å) and were included in the refinement in the riding model approximation, with *U*(H) set to 1.2*U*_eq_(C).

### Crystal data

**Table:**

C_21_H_16_N_2_O_3_	*F*(000) = 720
*M**_r_* = 344.36	*D*_x_ = 1.388 Mg m^−^^3^
Monoclinic, *P*2_1_/*c*	Mo *K*α radiation, λ = 0.71073 Å
Hall symbol: -P 2ybc	Cell parameters from 4815 reflections
*a* = 7.8539 (2) Å	θ = 2.2–29.4°
*b* = 11.5822 (3) Å	µ = 0.09 mm^−^^1^
*c* = 18.1455 (4) Å	*T* = 293 K
β = 93.537 (1)°	Block, orange
*V* = 1647.47 (7) Å^3^	0.40 × 0.35 × 0.20 mm
*Z* = 4	

### Data collection

**Table:**

Bruker X8 APEXII area-detector diffractometer	3053 reflections with *I* > 2σ(*I*)
Radiation source: fine-focus sealed tube	*R*_int_ = 0.039
graphite	θ_max_ = 30.0°, θ_min_ = 2.1°
φ and ω scans	*h* = −11→11
22612 measured reflections	*k* = −16→16
4806 independent reflections	*l* = −25→25

### Refinement

**Table:**

Refinement on *F*^2^	Secondary atom site location: difference Fourier map
Least-squares matrix: full	Hydrogen site location: inferred from neighbouring sites
*R*[*F*^2^ > 2σ(*F*^2^)] = 0.049	H-atom parameters constrained
*wR*(*F*^2^) = 0.153	*w* = 1/[σ^2^(*F*_o_^2^) + (0.0755*P*)^2^ + 0.2066*P*] where *P* = (*F*_o_^2^ + 2*F*_c_^2^)/3
*S* = 1.02	(Δ/σ)_max_ = 0.001
4805 reflections	Δρ_max_ = 0.31 e Å^−^^3^
236 parameters	Δρ_min_ = −0.22 e Å^−^^3^
0 restraints	Extinction correction: *SHELXL97* (Sheldrick, 2008), Fc^*^=kFc[1+0.001xFc^2^λ^3^/sin(2θ)]^-1/4^
Primary atom site location: structure-invariant direct methods	Extinction coefficient: 0.0033 (11)

### Fractional atomic coordinates and isotropic or equivalent isotropic displacement parameters (Å^2^)

**Table:**

	*x*	*y*	*z*	*U*_iso_*/*U*_eq_	
O1	0.14067 (16)	0.31774 (10)	0.53905 (6)	0.0588 (3)	
O2	0.23915 (17)	0.77316 (10)	0.53322 (7)	0.0623 (3)	
O3	0.44816 (18)	0.19062 (10)	0.30395 (6)	0.0633 (4)	
N1	0.35081 (16)	0.29295 (10)	0.40381 (6)	0.0405 (3)	
N2	0.42897 (16)	0.38962 (10)	0.30579 (6)	0.0422 (3)	
C1	0.32994 (17)	0.40923 (11)	0.41903 (7)	0.0342 (3)	
C2	0.27111 (16)	0.47213 (11)	0.47863 (7)	0.0342 (3)	
C3	0.18650 (18)	0.41799 (12)	0.54058 (7)	0.0387 (3)	
C4	0.14672 (17)	0.49192 (13)	0.60472 (8)	0.0408 (3)	
C5	0.0848 (2)	0.43992 (15)	0.66656 (9)	0.0532 (4)	
H5	0.0729	0.3601	0.6683	0.064*	
C6	0.0408 (2)	0.50686 (19)	0.72553 (9)	0.0635 (5)	
H6	−0.0001	0.4719	0.7671	0.076*	
C7	0.0572 (2)	0.62479 (19)	0.72301 (10)	0.0649 (5)	
H7	0.0265	0.6693	0.7627	0.078*	
C8	0.1187 (2)	0.67763 (16)	0.66225 (10)	0.0557 (4)	
H8	0.1301	0.7575	0.6610	0.067*	
C9	0.16416 (18)	0.61104 (13)	0.60235 (8)	0.0427 (3)	
C10	0.22782 (18)	0.66824 (13)	0.53631 (8)	0.0428 (3)	
C11	0.27960 (17)	0.59389 (12)	0.47464 (7)	0.0372 (3)	
C12	0.3362 (2)	0.64983 (13)	0.41289 (8)	0.0443 (3)	
H12	0.3403	0.7301	0.4122	0.053*	
C13	0.38660 (19)	0.58851 (12)	0.35255 (8)	0.0437 (3)	
H13	0.4219	0.6261	0.3108	0.052*	
C14	0.38263 (17)	0.47023 (12)	0.35652 (7)	0.0368 (3)	
C15	0.4124 (2)	0.28065 (13)	0.33395 (8)	0.0451 (4)	
C16	0.4861 (2)	0.40982 (14)	0.23155 (7)	0.0462 (4)	
H16A	0.5829	0.3603	0.2237	0.055*	
H16B	0.5234	0.4893	0.2277	0.055*	
C17	0.3483 (2)	0.38657 (17)	0.17310 (9)	0.0595 (5)	
H17	0.3070	0.3114	0.1688	0.071*	
C18	0.2826 (3)	0.4620 (2)	0.12858 (11)	0.0851 (7)	
H18A	0.3206	0.5380	0.1312	0.102*	
H18B	0.1969	0.4409	0.0935	0.102*	
C19	0.3537 (2)	0.18926 (12)	0.45100 (8)	0.0431 (3)	
H19A	0.3615	0.2133	0.5023	0.052*	
H19B	0.4552	0.1448	0.4424	0.052*	
C20	0.2018 (2)	0.11366 (14)	0.43818 (8)	0.0498 (4)	
H20	0.0939	0.1461	0.4403	0.060*	
C21	0.2140 (3)	0.00335 (17)	0.42404 (11)	0.0695 (5)	
H21A	0.3207	−0.0307	0.4217	0.083*	
H21B	0.1158	−0.0411	0.4163	0.083*	

### Atomic displacement parameters (Å^2^)

**Table:**

	*U*^11^	*U*^22^	*U*^33^	*U*^12^	*U*^13^	*U*^23^
O1	0.0796 (8)	0.0408 (6)	0.0591 (7)	−0.0081 (6)	0.0298 (6)	−0.0009 (5)
O2	0.0821 (9)	0.0354 (6)	0.0711 (8)	−0.0067 (6)	0.0191 (6)	−0.0114 (5)
O3	0.1046 (10)	0.0388 (6)	0.0494 (7)	0.0101 (6)	0.0285 (6)	−0.0027 (5)
N1	0.0577 (7)	0.0304 (6)	0.0343 (6)	0.0046 (5)	0.0110 (5)	0.0026 (4)
N2	0.0573 (7)	0.0374 (6)	0.0330 (6)	0.0023 (5)	0.0113 (5)	0.0013 (5)
C1	0.0386 (7)	0.0309 (6)	0.0332 (6)	0.0013 (5)	0.0033 (5)	0.0014 (5)
C2	0.0356 (7)	0.0342 (7)	0.0327 (6)	0.0008 (5)	0.0021 (5)	0.0006 (5)
C3	0.0418 (7)	0.0374 (7)	0.0373 (7)	0.0022 (6)	0.0067 (6)	0.0014 (6)
C4	0.0381 (7)	0.0484 (8)	0.0363 (7)	0.0042 (6)	0.0048 (6)	−0.0015 (6)
C5	0.0589 (10)	0.0575 (10)	0.0447 (8)	0.0056 (8)	0.0151 (7)	0.0033 (7)
C6	0.0697 (11)	0.0793 (13)	0.0434 (9)	0.0053 (10)	0.0189 (8)	−0.0030 (9)
C7	0.0712 (12)	0.0783 (14)	0.0465 (9)	0.0071 (10)	0.0143 (8)	−0.0192 (9)
C8	0.0608 (10)	0.0552 (10)	0.0518 (9)	0.0046 (8)	0.0077 (8)	−0.0152 (8)
C9	0.0399 (7)	0.0472 (8)	0.0409 (7)	0.0030 (6)	0.0024 (6)	−0.0077 (6)
C10	0.0443 (8)	0.0376 (8)	0.0466 (8)	−0.0008 (6)	0.0034 (6)	−0.0079 (6)
C11	0.0395 (7)	0.0330 (7)	0.0392 (7)	0.0004 (5)	0.0028 (6)	−0.0015 (5)
C12	0.0560 (9)	0.0311 (7)	0.0462 (8)	−0.0028 (6)	0.0064 (7)	0.0023 (6)
C13	0.0548 (9)	0.0366 (7)	0.0404 (7)	−0.0025 (6)	0.0087 (6)	0.0061 (6)
C14	0.0408 (7)	0.0361 (7)	0.0339 (7)	0.0010 (6)	0.0052 (5)	0.0013 (5)
C15	0.0618 (9)	0.0382 (8)	0.0365 (7)	0.0053 (7)	0.0123 (7)	0.0003 (6)
C16	0.0564 (9)	0.0479 (8)	0.0360 (7)	−0.0006 (7)	0.0158 (6)	0.0019 (6)
C17	0.0686 (11)	0.0708 (12)	0.0406 (8)	−0.0083 (9)	0.0168 (8)	−0.0026 (8)
C18	0.0780 (14)	0.131 (2)	0.0468 (10)	0.0076 (13)	0.0107 (9)	0.0108 (12)
C19	0.0573 (9)	0.0331 (7)	0.0394 (7)	0.0057 (6)	0.0076 (6)	0.0057 (6)
C20	0.0611 (10)	0.0430 (8)	0.0458 (8)	0.0004 (7)	0.0067 (7)	0.0062 (7)
C21	0.0869 (13)	0.0486 (10)	0.0739 (13)	−0.0104 (10)	0.0114 (10)	−0.0024 (9)

### Geometric parameters (Å, °)

**Table:**

O1—C3	1.2154 (18)	C8—H8	0.93
O2—C10	1.2200 (18)	C9—C10	1.483 (2)
O3—C15	1.2170 (17)	C10—C11	1.4884 (19)
N1—C1	1.3867 (16)	C11—C12	1.3908 (19)
N1—C15	1.3918 (18)	C12—C13	1.383 (2)
N1—C19	1.4742 (17)	C12—H12	0.93
N2—C15	1.3708 (19)	C13—C14	1.372 (2)
N2—C14	1.3757 (17)	C13—H13	0.93
N2—C16	1.4647 (17)	C16—C17	1.493 (2)
C1—C2	1.4056 (18)	C16—H16A	0.97
C1—C14	1.4194 (18)	C16—H16B	0.97
C2—C11	1.4139 (19)	C17—C18	1.277 (3)
C2—C3	1.4800 (18)	C17—H17	0.93
C3—C4	1.4934 (19)	C18—H18A	0.93
C4—C9	1.387 (2)	C18—H18B	0.93
C4—C5	1.388 (2)	C19—C20	1.486 (2)
C5—C6	1.382 (2)	C19—H19A	0.97
C5—H5	0.93	C19—H19B	0.97
C6—C7	1.373 (3)	C20—C21	1.308 (2)
C6—H6	0.93	C20—H20	0.93
C7—C8	1.375 (3)	C21—H21A	0.93
C7—H7	0.93	C21—H21B	0.93
C8—C9	1.397 (2)		
			
C1—N1—C15	109.44 (11)	C2—C11—C10	121.50 (12)
C1—N1—C19	132.32 (11)	C13—C12—C11	121.32 (13)
C15—N1—C19	116.86 (11)	C13—C12—H12	119.3
C15—N2—C14	109.90 (11)	C11—C12—H12	119.3
C15—N2—C16	122.09 (12)	C14—C13—C12	117.55 (13)
C14—N2—C16	128.00 (12)	C14—C13—H13	121.2
N1—C1—C2	134.84 (12)	C12—C13—H13	121.2
N1—C1—C14	106.28 (11)	C13—C14—N2	129.39 (13)
C2—C1—C14	118.87 (12)	C13—C14—C1	123.19 (13)
C1—C2—C11	117.30 (12)	N2—C14—C1	107.40 (12)
C1—C2—C3	123.31 (12)	O3—C15—N2	126.33 (14)
C11—C2—C3	119.10 (12)	O3—C15—N1	126.71 (14)
O1—C3—C2	122.25 (13)	N2—C15—N1	106.95 (12)
O1—C3—C4	119.32 (13)	N2—C16—C17	112.01 (13)
C2—C3—C4	118.31 (12)	N2—C16—H16A	109.2
C9—C4—C5	119.78 (14)	C17—C16—H16A	109.2
C9—C4—C3	121.35 (13)	N2—C16—H16B	109.2
C5—C4—C3	118.81 (14)	C17—C16—H16B	109.2
C6—C5—C4	119.96 (17)	H16A—C16—H16B	107.9
C6—C5—H5	120.0	C18—C17—C16	125.0 (2)
C4—C5—H5	120.0	C18—C17—H17	117.5
C7—C6—C5	120.24 (17)	C16—C17—H17	117.5
C7—C6—H6	119.9	C17—C18—H18A	120.0
C5—C6—H6	119.9	C17—C18—H18B	120.0
C6—C7—C8	120.54 (16)	H18A—C18—H18B	120.0
C6—C7—H7	119.7	N1—C19—C20	113.92 (13)
C8—C7—H7	119.7	N1—C19—H19A	108.8
C7—C8—C9	119.83 (18)	C20—C19—H19A	108.8
C7—C8—H8	120.1	N1—C19—H19B	108.8
C9—C8—H8	120.1	C20—C19—H19B	108.8
C4—C9—C8	119.64 (15)	H19A—C19—H19B	107.7
C4—C9—C10	120.54 (13)	C21—C20—C19	122.60 (17)
C8—C9—C10	119.81 (15)	C21—C20—H20	118.7
O2—C10—C9	120.76 (13)	C19—C20—H20	118.7
O2—C10—C11	121.17 (14)	C20—C21—H21A	120.0
C9—C10—C11	118.07 (13)	C20—C21—H21B	120.0
C12—C11—C2	121.62 (13)	H21A—C21—H21B	120.0
C12—C11—C10	116.87 (13)		
			
C15—N1—C1—C2	178.83 (15)	C1—C2—C11—C10	−177.23 (12)
C19—N1—C1—C2	−15.3 (3)	C3—C2—C11—C10	8.7 (2)
C15—N1—C1—C14	−0.69 (16)	O2—C10—C11—C12	−1.9 (2)
C19—N1—C1—C14	165.13 (14)	C9—C10—C11—C12	179.00 (13)
N1—C1—C2—C11	176.13 (15)	O2—C10—C11—C2	178.30 (14)
C14—C1—C2—C11	−4.39 (18)	C9—C10—C11—C2	−0.8 (2)
N1—C1—C2—C3	−10.1 (2)	C2—C11—C12—C13	0.1 (2)
C14—C1—C2—C3	169.38 (12)	C10—C11—C12—C13	−179.75 (14)
C1—C2—C3—O1	−10.6 (2)	C11—C12—C13—C14	−1.6 (2)
C11—C2—C3—O1	163.09 (14)	C12—C13—C14—N2	−178.38 (14)
C1—C2—C3—C4	173.42 (12)	C12—C13—C14—C1	−0.1 (2)
C11—C2—C3—C4	−12.91 (19)	C15—N2—C14—C13	176.95 (15)
O1—C3—C4—C9	−166.55 (14)	C16—N2—C14—C13	−3.9 (3)
C2—C3—C4—C9	9.6 (2)	C15—N2—C14—C1	−1.58 (16)
O1—C3—C4—C5	10.9 (2)	C16—N2—C14—C1	177.55 (14)
C2—C3—C4—C5	−172.99 (13)	N1—C1—C14—C13	−177.27 (13)
C9—C4—C5—C6	0.0 (2)	C2—C1—C14—C13	3.1 (2)
C3—C4—C5—C6	−177.51 (15)	N1—C1—C14—N2	1.38 (15)
C4—C5—C6—C7	0.4 (3)	C2—C1—C14—N2	−178.24 (12)
C5—C6—C7—C8	−0.5 (3)	C14—N2—C15—O3	−177.54 (17)
C6—C7—C8—C9	0.3 (3)	C16—N2—C15—O3	3.3 (3)
C5—C4—C9—C8	−0.2 (2)	C14—N2—C15—N1	1.15 (17)
C3—C4—C9—C8	177.27 (14)	C16—N2—C15—N1	−178.04 (13)
C5—C4—C9—C10	−179.13 (14)	C1—N1—C15—O3	178.43 (16)
C3—C4—C9—C10	−1.7 (2)	C19—N1—C15—O3	10.1 (3)
C7—C8—C9—C4	0.0 (2)	C1—N1—C15—N2	−0.26 (18)
C7—C8—C9—C10	178.99 (15)	C19—N1—C15—N2	−168.54 (12)
C4—C9—C10—O2	178.08 (14)	C15—N2—C16—C17	76.63 (19)
C8—C9—C10—O2	−0.9 (2)	C14—N2—C16—C17	−102.40 (18)
C4—C9—C10—C11	−2.8 (2)	N2—C16—C17—C18	116.73 (19)
C8—C9—C10—C11	178.24 (14)	C1—N1—C19—C20	109.60 (18)
C1—C2—C11—C12	2.9 (2)	C15—N1—C19—C20	−85.41 (17)
C3—C2—C11—C12	−171.10 (13)	N1—C19—C20—C21	126.87 (17)

### Hydrogen-bond geometry (Å, °)

**Table:**

*D*—H···*A*	*D*—H	H···*A*	*D*···*A*	*D*—H···*A*
C13—H13···O3^i^	0.93	2.49	3.406 (2)	168
C16—H16B···O3^i^	0.97	2.42	3.362 (2)	165

Symmetry codes: (i) −*x*+1, *y*+1/2, −*z*+1/2.
